# Feasibility Analysis on the Adoption of Decentralized Anaerobic Co-Digestion for the Treatment of Municipal Organic Waste with Energy Recovery in Urban Districts of Metropolitan Areas

**DOI:** 10.3390/ijerph18041820

**Published:** 2021-02-13

**Authors:** Giovanni Gadaleta, Sabino De Gisi, Michele Notarnicola

**Affiliations:** Dipartimento di Ingegneria Civile, Ambientale, del Territorio, Edile e di Chimica, Politecnico di Bari, Via E. Orabona, 4, I-70125 Bari, Italy; giovanni.gadaleta@poliba.it (G.G.); michele.notarnicola@poliba.it (M.N.)

**Keywords:** anaerobic digestion, co-generation, decentralized system, methane yield, municipal solid waste, techno-economic analysis

## Abstract

Anaerobic digestion (AD) of organic fraction of municipal solid waste (OFMSW) is considered an excellent solution for both waste management and energy generation, although the impacts of waste collection and transportation on the whole management system are not negligible. AD is often regarded as a centralized solution for an entire community, although recently, there has been some debate on the adoption of decentralized, smaller facilities. This study aims to evaluate the techno-economic feasibility of an AD plant at the local scale for the treatment of organic waste generated from urban districts. Depending on the type of feedstock, two scenarios were evaluated and compared with the reference scenario, based on composting treatment: (1) mono-AD of OFMSW and (2) co-AD of OFMSW and sewage sludge (SS). Furthermore, different district extensions of the metropolitan area were considered with the goal of determining the optimal size. Results showed the advantage of the two scenarios over the reference one. Scenario 1 proved to be the most suitable solution, because the introduction of SS in Scenario 2 increased costs and payback time, rather than generating a higher waste amount and lower biogas yield. The preferred district extension was the medium-sized one. Capital cost strongly affected the economic analysis, but revenue from the city for the management operation of the organic waste could significantly decrease costs. Further studies about the differences in the type of feedstock or the introduction of other criteria of analysis (such as environmental) are considered necessary.

## 1. Introduction

Every year, in Italy, around 30 million tons of municipal solid waste (MSW) are generated, and about half of this amount is represented by organic biodegradable waste, also known as the organic fraction of MSW (OFMSW) [1]. OFMSW is mainly composed of food waste (FW) from domestic kitchens and yard waste (YW) from garden maintenance. Due to a high carbon and volatile solid (VS) content, the first is considered as a potential energy source [2], and the latter comprises green waste, such as grass, leaves, and branches, characterized by high lignin content [3]. Currently, the main OFMSW treatment systems are composting and anaerobic digestion (AD). Composting is a biological treatment that aerobically decomposes organic waste, producing compost with a release of heat, humidity, and carbon dioxide (CO_2_). Compost is a nutrient-rich soil that can be used as an agricultural amendment. AD is an anaerobic biological treatment for different types of substrates that converts organic matter to biogas and digestate [4]. Biogas consists of 50–70% methane (CH_4_), 25–45% CO_2_, and traces of hydrogen sulfide (H_2_S), humidity, and other gases, thus, it can be used as a source for renewable electric and thermal energy production through a combined heat and power (CHP) system [5]. Digestate is rich in nutrients, but, due to a lower waste biodegradation of the AD treatment than composting, it doesn’t achieve good biological stability, and requires further treatments [6]. AD treatment is receiving increasing attention due to its high valorization value for waste; FW presents an excellent organics degradation rate and methane productivity, and YW contains a compact structure that impedes the attack of anaerobes, thus often resulting in a long digestion period and low biogas yield [7]. Hence, by balancing the characteristics of FW and YW substrates in OFMSW, anaerobic co-digestion (co-AD) of OFMSW could be considered a significant strategy for the management of these wastes in terms of circular economy and performance.

Generally, waste management is a difficult practice, and often includes elevated costs. In Italy, the costs of waste collection and disposal services covers the main part of the funding for waste management [8]. Other than the environmental pressure exerted by waste treatment plants, the effects of waste transportation from the collection points are not negligible, compared to those generated by other segments of the waste management system [9]. Concerning the waste management through AD, Lamnatou et al. [10] showed that the transportation of organic waste impacts from 44% to over 70% of the whole environment. Hence, the introduction of a transfer station or small waste treatment plant with a view to a decentralized system, instead of a bigger one, could decrease environmental impacts, such as energy demand and emissions, especially in medium and large cities [11]. Despite the economic cost required by new infrastructures, the introduction of an AD plant at a local site could reduce the cost of waste transportation and generate energy from biogas combustion. Energy could be delivered to the urban electricity network or on-site to compensate for the energy consumption of the AD plant [12]. Similar plants exist around the world, and sparse proof-of-concepts can be found in the literature. Walker et al. [13] have presented an urban micro-scale AD plant located in London (UK). The plant was built in 2013 and continues to operate to-date, processing urban food waste and generating biogas for use in a community café.

While co-AD is technically and scientifically widely reported in the literature, most of the studies focused on OFMSW treatment and management at a local site are still few in number, and results are often in conflict. The deployment of a medium-sized biogas power plant from OFMSW was promoted by Di Matteo et al. [14]. Three urban models were identified and related with a typical OFMSW matrix for each urban area. The OFMSW types had a significant effect on the energy production, and source separated OFMSW gave the best results. Different scenarios based on AD treatment of OFMSW were assessed through a life cycle assessment by Grosso et al. [15], and the results were compared to the real metropolitan situation, where food waste ended up mixed with the residual waste in a waste-to-energy plant. All new scenarios based on AD attained similar or better results compared with the reference one for almost all of the impact indicators. Mainardis et al. [16] evaluated the techno-economic feasibility of AD implementation in small breweries located in northern Italy. Due to good biodegradability and high-energy content of feedstock, energy balance revealed that AD implementation could be a sustainable solution for providing most of the energy needed. From an economic point of view, the feasibility of commercial-scale AD and composting systems that received both YW and liquid AD effluent has been evaluated by Lin et al. [17]. The results suggest that AD would be more economically favorable than composting when the plant size increases. Therefore, AD and composting may be favored for centralized and de-centralized treatment, respectively. Furthermore, micro-composting of OFMSW in residential areas was investigated by Chanakya et at. [18]. The suitability of these substrates for micro-composting in plastic bins was evaluated by tracking the decomposition pattern and physical changes. All of the feedstock analyzed was found to have good biological methane potential, and showed promise for conversion to biogas under a mixed feed operation. Thus, AD appears to be a more suitable micro treatment option.

From the literature review, it was revealed that only a few publications have reported the analysis of an AD plant at the local scale; furthermore, no study has investigated the application of a co-AD plant in the urban districts of large cities for the management of domestic substrates (as OFMSW) as a renewable resource of energy for urban need.

Hence, the aim of this work was to evaluate the technical and economic feasibility of a co-AD plant at the local scale for energy production. For this purpose, the co-AD process was studied considering as a substrate OFMSW for Scenario 1, and a mixture of OFMSW and sewage sludge (SS) from a municipal wastewater treatment plant (MWWTP) for Scenario 2. Five different levels of district extension in terms of the number of buildings were considered. Methane yield of the mixture, energy consumption by the plant, and net energy produced were calculated through a simplified scheme. The levelized cost of waste (LCOW) index was estimated in order to evaluate the best district extension in economic terms. Each scenario result was compared with the reference scenario (Scenario 0) based on organic waste collection and composting.

## 2. Materials and Methods

To assess how the district extension affects the feasibility of a co-AD plant at the local scale, this work presented a methodological approach by a parametric analysis, along with the support of data from the literature. The key elements of the analysis were (i) the urban district; (ii) the substrates properties; (iii) the co-AD plant parameters; and (iv) the CHP system. After deep consulting of different literature works, average values in line with the literature have been considered for this study. These elements were used for the calculation of the LCOW index in order to carry out a comprehensive economic analysis.

### 2.1. Scenario Definition

**Figure 1:**
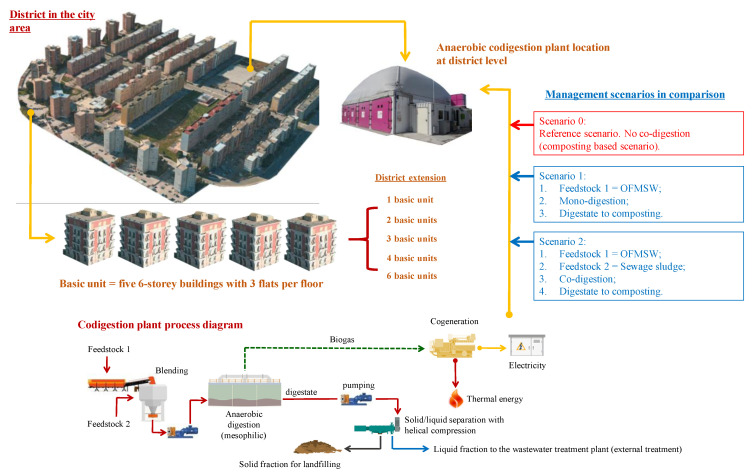
Management scenarios in comparison.

Two scenarios were defined. The first was referred to a mono-AD plant that used the municipal OFMSW produced by the districts (Scenario 1). The second considered the possibility of implementing the mono-AD plant in the co-digestion of SS from MWWTP (co-AD) in addition to the existing OFMSW stream (Scenario 2). This possibility explored the fact that small districts produce a modest amount of SS that can be easily used in the AD process, avoiding their management in a dedicated MWWTP. Both scenarios were evaluated for different district extensions, and then compared with the reference scenario, characterized by a waste management without the implementation of mono- or co-AD plants for the urban district (Scenario 0). In this case, all of the organic waste produced is collected separately and sent to a typical Italian composting plant. Figure 1 sketches district extensions and treatment scenarios.

### 2.2. Urban Model

The extension of the urban district was represented by the number of condominiums (Nc) that composed each district. Five different district extensions were evaluated, assuming the value of 5, 10, 15, 20, and 30 for Nc. The total number of people (Np_TOT_) of the district was evaluated using the description of the building and demographic characteristics of the proposed condominium (Table 1) through Equation (1).

Np_TOT_ = Nc · Nu · Nf · Na · Np, (1)

The values in Table 1 are characteristics of buildings in southern Italy. In particular, it has been assumed that the mean of the people that compose a family and live in an apartment is equal to 2.6 [19].

Due to the different lifestyles and habits of people, the daily MSW production per capita and the organic content could differ greatly according to the economic prosperity of the country analyzed. In most of the developing countries, the OFMSW represents half of this amount [20]. Therefore, in this study, the calculation of the OFMSW amount was obtained considering an MSW production of 1.2 kg MSW/capita/d and an organic content of 50%, the typical value in the context of southern Italy [21]. In addition, OFMSW was mainly composed of FW and YW [1]. Thus, the OMSW stream generated by the people considered (OFMSW_in_) can be calculated as follows (Equation (2)):

OMSW_in_ = Np_TOT_ · 1.2 · 0.5, (2)

Due to the variability of FW and YW generation [22], the OFMSW composition is taken as the average of the literature data.

The estimation of the daily SS stream (SS_in_) was carried out assuming two different MWWTP schemes. In increasing the size of the served city (and, consequently, the number of people), the process scheme became more articulated. It has been assumed that when Np_TOT_ is less than 10,000 units, the MWWTP is composed of a primary sedimentation for the water line and a thickening unit for the sludge line. In this case, SS was composed of only primary sludge (PS). Instead, when Np_TOT_ was more than 10,000 units, an MWWTP layout with primary and secondary sedimentation for the water line and a centrifugal unit for the dewatering process of the sludge line was assumed. In this second case, SS was composed of PS and waste activated sludge (WAS). In order to quantify the SS production, 0.050 kg/capita/d and 0.0025 kg/capita/d were assumed for the SS daily amount per capita (SSda_i_) of PS and WAS, respectively [23]. Finally, SS_in_ was calculated through Equation (3):

SS_in_ = (Np_TOT_ · SSda_PS_ · X_PS_) · (Np_TOT_ · SSda_WAS_ · X_WAS_), (3)

In order to evaluate both of the SS types (composed of PS and WAS or only PS when Np_TOT_ > 10,000 and Np_TOT_ < 10,000, respectively), the X_i_ parameter was used as a binary variable (1–0). All of the parameters assumed for the SS_in_ calculation are summarized in Table 2.

Hence, the waste generated daily by the whole system (W_in_) was the sum of each waste stream, as determined through Equation (4):

W_in_ = OFMSW_in_ + SS_in_, (4)

### 2.3. Substrates

FW, YW, and SS examined in other studies were characterized in terms of total solid (TS) and volatile solid (VS) content, bio-methane potential (BMP), and methane content of the biogas, reported in Table 3.

The input streams to the co-AD plant were OFMSWW and SS. OFMSW was mainly composed of FW and YW; FW was a typical source-separated domestic kitchen and canteen food waste, and YW represented all of the waste produced by the pruning and maintenance operations of public gardens or private gardening [43]. SS described a specified mixture of primary sludge (PS) and waste activated sludge (WAS) from an MWWTP. The properties of OFMSW, summarized in Table 4, were assumed constant for all of the scenarios and municipal district extensions analyzed.

Depending on the MWWTP scheme adopted, the properties and types of SS varied (Table 2). As described earlier, the district extension affects the MWWTP type. Under 10,000 people, there was only PS, while above 10,000, there was a mixture of PS and WAS. In the first case, PS assumed the values of 5.5% and 300 mL CH_4_/g VS for TS content and BMP, respectively. In the latter, PS was characterized by a higher TS value (25%), but a lower BMP (125 mL CH_4_/g VS). This condition depends on the fact that SS with a lower bulk density was characterized by a higher BMP value (Table 2). In fact, the adoption of a centrifugal unit despite a thickening one could achieve a more efficient dewatering process [44]. The properties of the PS-WAS mixture was assumed to be the weighted average of each stream that composed it. Thus, the same weighted average was calculated for defining the W_in_ stream properties.

### 2.4. AD Reactor

Assumed to be located in southern Italy, the mono- and co-AD plant scheme was based on a typical Italian AD plant [45]. Depending on the scenario, the digester was daily fed with a mixture of OFMSW and SS in a certain proportion. It was assumed that the digester worked in a mesophilic temperature (35 °C); the process was conducted with a hydraulic retention time (HRT) of 30 d [26]. The volume V of the digester was calculated using the density (d) of the waste input and the HRT value through Equation (5):

V = 1.2 · W_in_ · HRT/(d · 1000), (5)

The value thus obtained was increased by 20% in order to avoid digester filling. The volume value aimed to show an indicative value: it has to be adjusted according to a design that takes into account the condition of the process (wet or dry anaerobic digestion).

The biodegradation process that took place in the digester reduced by 90% the inlet VS stream. The biodegradation is strictly dependent on the SS/OFMSW ratio [46]. Hence, for Scenario 2, a VS removal value that depended on the SS amount according to Gu et al. [42] was assumed, and instead, for Scenario 1, the value was assumed equal to 90%. The solid digestate was sent to a solid/liquid separation by helical compression. As reported in previous works [44,47], this equipment achieved a water removal of 30%. Considering the VS and water removal during the biodegradation and the dewatering process, respectively, the output waste stream (W_out_) was calculated using Equation (6):

W_out_ = W_in_ − (W_in_ · TS · VS · 90%) − [W_in_ · (1 − TS) · 30%], (6)

It was assumed that the AD process did not achieve a biologically stable digestate, and, therefore, the dewatered digestate was finally treated in the same composting plant of Scenario 0.

On the other hand, the daily biogas production (B_out_) was evaluated using the BMP value of W_in_ stream, as showed by Equation (7):

B_out_ = W_in_ · TS · VS · BMP/(1000 · %CH_4_), (7)

The main characteristics of the digester and the dewatering system were reported in Table 5.

### 2.5. CHP

Biogas was sent to a CHP system with an internal combustion engine of 200 kW for the combined production of electricity and heat. In line with other CHP systems [12,32,52], the electrical (η_el_) and thermal (η_th_) CHP efficiency was assumed equal to 35% and 50%, respectively. According to Ghosh et al. [50], a lower heating value (LHV) of CH_4_ equal to 35.25 MJ/m^3^ was assumed. Due to the non-reactive properties of CO_2_ in the combustion process of biogas, the biogas LHV was calculated through Equation (8):

LHV_biog_ = LHV_CH4_ · %CH_4_, (8)

Hence, the daily gross energy (gE) generation in terms of kWh/d could be calculated through Equations (9) and (10) for the electric and thermal energy, respectively. Consequently, the energy loss due to the transmission and generation of electrical and thermal energy could be calculated through Equation (11):

gE_el_ = LHV_biog_ · B_out_ · η_el_, (9)

gE_th_ = LHV_biog_ · B_out_ · η_th_, (10)

gE_loss_ = LHV_biog_ · B_out_ · (1 − η_el_ − η_th_), (11)

The net energy was evaluated by subtracting the self-consumption need of the co-AD, dewatering, and CHP systems from gE. The electrical energy was reduced due to the demand for energy for the pumping, mixing, automatic control system, CHP operation, dewatering system, and shredding pretreatment. The thermal energy was reduced due to the energy request for the digester temperature maintenance. In line with previous studies [12,32], the self-consumption values assumed in this work were 68 kWh/t and 40 kWh/t for electrical and thermal energy, respectively. The first ones could be further divided in 60 kWh/t and 8 kWh/t for pretreatments and an operative energy request, respectively [32,48,51]. The electricity requirements of the dewatering system, which was assumed to be 10 kWh/t [49], had to be subtracted from gE_el_. Hence, the amount of the net electrical (nE_el_) and thermal (nE_th_) energy could be formulated as follows by Equations (12) and (13), respectively:

nE_el_ = gE_el_ − [W_in_ · (68+10)/1000], (12)

nE_th_ = gE_th_ − [W_in_ · 40/1000], (13)

In order to evaluate the CHP capacity (N_CHP_), the number of hours (Nh) that the unit will work was estimated through Equation (14):

Nh = nE_el_/(200 · N_CHP_), (14)

If Nh was more than 24 h, N_CHP_ was equal to 2, and otherwise, N_CHP_ was equal to 1. Thus, the denominator of Equation (14) represented the total CHP capacity. The main characteristics of the CHP unit and the biogas properties are reported in Table 5.

### 2.6. The LCOW

The financial feasibility of the project has been evaluated with the LCOW index. This index measured the average net present cost of a specific waste management system for a waste source over its lifetime. The LCOW was calculated as the sum of all of the discounted net costs over the lifetime of a waste management scenario divided by a discounted sum of the waste amounts generated. The inputs to the LCOW were capital cost, operative and maintenance costs, treatment costs, and revenues from thermal and electrical energy. Similar indexes were used by Wang et al. [53] and Carlini et al. [51]. Thus, the LCOW referred to a single scenario, and it was calculated through Equation (15):

(15) $$\mathrm{LCOW}\;=\;\frac{\sum_{t=1}^{n}\frac{C_{t}+O_{t}+T_{t}-Re_{t}-Rt_{t}}{{\left(1+r\right)}^{t}}}{\sum_{t=1}^{n}\frac{W_{in}}{{\left(1+r\right)}^{t}}},$$

where *C_t_* is the capital cost at the year *t*, *O_t_* is the operative cost at the year *t*, T*_t_* is the waste treatment cost at the year *t*, *Re_t_* and *Rt_t_* are the revenues at the year *t* from the sale of electrical and thermal energy, respectively, *r* is the discount rate, and *t* is the expected lifetime of the waste management system. *C_t_* involved all of the costs related to the installation of the waste management system, as well as the purchase of the machines. According to previous studies [33,53], it was assumed equal to 4000 €/kW. In line with the literature [29,49,53], *O_t_* involved all of the operative and maintenance costs, and it was assumed equal to 105 €/t of treated waste. *T_t_* was the cost for the waste treatment in the composting plant of the digestate in output from the mono- and co-AD plant for Scenarios 1 and 2, respectively, or the generated waste for Scenario 0. This cost was assumed equal to 75 €/t, and the value involved the operative and administrative costs of the plant. The value was in line with the regional MSW management plan [54], as the cost of an Italian composting plant [55]. In Scenario 0, the collection cost was added to the treatment one. The collection cost was 10 €/capita, as reported in the Italian waste management report [1]. Revenues were estimated considering that, in Italy, the price is different if the energy is produced by renewable biomasses [26]. Despite the existence of different tariffs [51], the typical Italian values of 0.2 €/kWh and 0.045 €/kWh for the energy and thermal energy selling, respectively, did not differ from those reported in the literature [33,52,53]. The *r* and *t* values were 4% and 20 years, respectively [51,52,53]. Table 6 summarizes all of the parameters involved for the LCOW calculation.

The calculation of costs and revenues for each scenario gave the possibility of knowing the payback time (PBT) of the digester where PBT indicated the time, in terms of years, required for achieving a profit equal to the initial capital cost.

## 3. Results

### 3.1. Scenario 1

The properties of W_in_ stream for Scenario 1 are presented in Table 7.

The input waste achieved 40% of TS, which is the limit value for an ultra-dry mono-AD process [45], and 88.6% of TS of VS for each urban extension. Bulk density, BMP, and methane content of biogas were constant in each case, equal to 0.43 kg/L, 364.5 mL CH_4_/gVS, and 61.4%, respectively. The daily amount of waste generated increased linearly with the rising of Nc. As reported in Table 8, the minimum and maximum values of W_in_ were 3370 kg/d and 20,128 kg/d, respectively, which were composed only of OFMSW. The digester volume V, B_out_, and W_out_ streams are reported in Table 8.

V was between 284 and 1704 m^3^, which could be compared with a cube with a side from about 7 to 12 m. The OLR remained constant at 5 kgVS/m^3^/d. Incoming waste decreased by half due to the anaerobic biodegradation of the OFMSW and the dewatering process. As a result, W_out_ stream achieved a TS and VS content of 16% and 44% TS, respectively. B_out_ increased from 711 m^3^/d to 4266 m^3^/d with a linear trend: no biogas storage was operated, but it was sent directly to the CHP unit for the energy conversion. Hence, from an energetic point of view, the net electrical and thermal energy generated by the plant are presented in Table 9.

Results revealed that the plant generated more thermal energy than electricity, according to the higher electrical energy for self-consumption and a lower CHP efficiency. Self-consumptions were about the 10% of the gross energy generated by the plant. As reported in Table 10, over 15 condominiums to adopt a second CHP unit were necessary.

**Figure 2:**
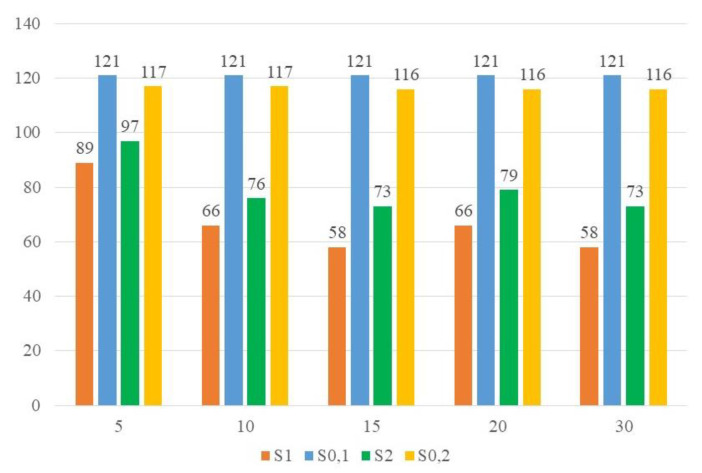
Results from the LCOW analysis of Scenario 0, 1, and 2, for Nc from 5 to 30.

The maximum working time of the unit was 22 h for both 15 and 30 Nc. Finally, the economic field was evaluated through the LCOW. Figure 2 shows the LCOW value for each district extension.

A decreasing trend was observed when Nc went from 5 to 15. The LCOW of 20 Nc was higher, but the value decreased again for 30 Nc. The best values were achieved by 15 and 30 Nc. 

**Figure 3:**
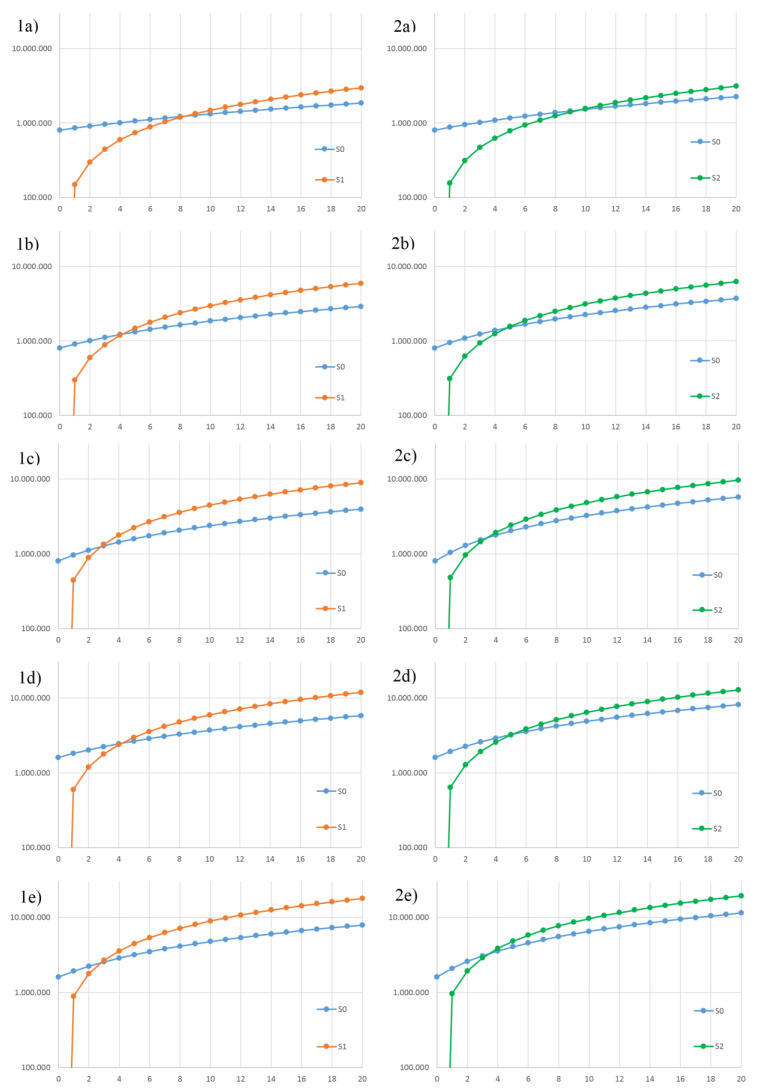
Results from the cumulative net cost analysis for Scenario 1 (**1**) and Scenario 2 (**2**), both compared to Scenario 0, for Nc equal to (**1a**,**1b**) 5, (**1b**,**2b**) 10, (**1c**,**2c**) 15, (**1d**,**2d**) 20 and (**1e**,**2e**) 30. Note that the *x* axis stands for project years and the *y* axis stands for cumulative net cost values (log scale).

Figure 3 summarizes the cumulated net costs of Scenario 1 and Scenario 0 for each urban extension.

For all of the cases, the net cumulated cost trend of Scenario 1 showed a first higher value, due to the capital cost that is null for Scenario 0, but not for Scenario 1 (from 800,000 to 1,600,000 €). The PBTs were less than 20 years for all of the district extensions (Table 10). Only for 5 Nc was the PBT near the digester lifetime. The best results in terms of PBT were given by 15 and 30 Nc, for which a PBT of six years was achieved.

### 3.2. Scenario 2

The introduction of SS in the OFMSW substrate modified the properties of the W_in_ stream, as shown in Table 7. The OFMSW content in the organic mixture decreased from 92.3% to 88.9%. The input waste reached final TS and VS values that were strictly dependent on the district extension. When Nc was less than 10, W_in_ showed a TS and VS content of 37.8–39.2% and 87.2% TS, respectively; when Nc increased, these values changed to 38.3% and 86.7% TS, respectively. The bulk density, BMP, and methane content of the biogas were also affected by this change. As a result of TS increase, the bulk density raised from 0.48 to 0.55 kg/l, while the decrease of VS made the BMP vary from 357.9 mL/gVS to 340.9 mL/gVS. The methane content appeared to vary slightly. The change in the daily amount and composition of SS affected the daily amount of waste generated. W_in_ lost the linear increasing with the rising of Nc, showing minimum and maximum values of 3650 kg/d and 22,745 kg/d, respectively (Table 8). V was between 274 and 1498 m^3^ (Table 8), which could be compared to a cube of a side from about 6 to 11 m. The OLR achieved a value between 5 and 6 kgVS/m^3^/d. The anaerobic degradation and the dewatering process reduced the W_in_ stream by a little less than 50%. As a result, the W_out_ stream achieved a TS and VS content of 15–16% and 41% TS, respectively. B_out_ increased from 700 m^3^/d to 4171 m^3^/d; no biogas storage was operated, but it was sent directly to the CHP unit for the energy conversion. nE_el_ and nE_th_ values (Table 9) confirmed the result of Scenario 1, in which the plant generated more thermal energy than electric. Self-consumptions were more than 10% of the gross energy generated by the plant. As shown in Table 10, N_CHP_ and Nh values were the same as in Scenario 1. The economic analysis through the LCOW index is reported in Figure 2. With the rising of urban extension, the LCOW values decreased until 15 Nc. Except for 20 Nc, where the LCOW was increased, the downward trend of the LCOW for 30 Nc continued. It is important to note that, contrary to Scenario 1, the LCOW values referred to 10 and 20 Nc were different. Again, the best LCOW values were achieved by 15 and 30 Nc. As reported in Figure 3, the net cumulated costs revealed a higher initial cost for Scenario 2, despite Scenario 0. Except for 5 Nc, where the PBT was near 20 years, all of the other district extensions showed PBT values near the half lifetime of the plant (Table 10).

## 4. Discussion

Starting from the W_in_ properties and characterization, the results from this study were in line with previous works. The W_in_ stream composed only of OFMSW (Scenario 1) had TS and VS values near to 39% and 92% TS, respectively, of a YW and FW mixture in a 1:3 proportion [7]. Scenario 1 values of W_in_ density, BMP, and methane content, respectively, were also confirmed by other works [12,20,21]. Instead, for Scenario 2, the SS BMP value was near 170 mL CH4/gVS, according to Shin et al. [38]. In addition, the W_in_ properties for Scenario 2 were similar to the ones achieved by a mixture of YW, FW, and SS (in a 3:9:4 proportion), characterized by TS, VS, methane content, and BMP values of 35.9%, 82.8% TS, 64.4%, and 314.9 mL CH4/gVS, respectively [12]. By focusing on Scenario 2, the W_in_ properties changed after 15 Nc as a result of the variation of SS characteristics and amounts. It is important to note that, despite the fact that the BMP assumed a lower value when Nc exceeded 15 units, the methane content remained unchanged.

From a technical point of view, the TS content of Scenario 1 was near the limit value of the ultra-dry process for AD. The introduction of SS (Scenario 2) led to a decrease in this value to a more comfortable one. As shown in Table 8, Scenario 2 was characterized by a higher W_in_ stream for each district extension. Despite this condition, in Scenario 2, V was always lower than in Scenario 1, due to a higher density value. On the other hand, the lower VS and BMP values of Scenario 2 led to a minor B_out_ stream. Thus, this study confirmed that the introduction of SS in OFMSW co-AD leads to decreased methane production compared with mono-digestion of OFMSW single substrates [12,42,46].

From an energetic point of view, the increasing in the W_in_ stream that characterized Scenario 2 could lead to a more major self-consumption need than Scenario 1. The electrical energy production per m^3^ of the biogas achieved a maximum value of about 2.1 for both Scenario 1 and 2, according to previous works [5,33,52]. Nh and N_CHP_ remained unchanged in both Scenario 1 and 2. The value of 200 kW per CHP unit was referred to a great capacity if compared to the typical literature value; almost 30% of Italian AD plants have a capacity between 100 and 500 kW [51]. Thus, the co-AD plant under examination could be compared to a medium-large industrial AD plant. The introduction of a biogas storage unit could permit the adoption of a lower CHP unit. Finally, the LCOW analysis revealed better results for district extensions of 15 and 30 Nc. The choice of 15 Nc could reduce the district extension, leading to an easier independent OFMSW delivery by people. The LCOW of Scenario 0 achieved a value of 121 and 117 for the scheme of Scenario 1 and 2, respectively. This showed that the introduction of SS in the inlet waste for composting could have a positive impact from an economic point of view. For Scenarios 1 and 2, the PBT for 15 Nc was near 4, referring to an AD plant with a 500 kW capacity [51]. A lower PBT could be obtained with a lower treatment cost of digestate [52]. It was important to note that the economic analysis did not involve the revenues paid by cities or urban hygiene service administrators served by the plant for organic waste management. These compensations depend on the contract between plant owners and the affiliates [56], and they are difficult to estimate. In addition, no revenues from user waste management taxes were considered. This kind of revenue could be significant in an economic analysis, and could lead to reduced costs until reaching a net gain. It is important to note that composting treatment was preferred to disposal in a landfill. Despite the fact that the inlet waste tariff of the landfill was lower than the composting one, the Italian government has introduced the “green tax” that the local authorities have to pay for waste disposal [57]. This value could increase the cost of landfilling to a higher value than the composting one.

From an economic point of view, governments of different countries have been promoting biomass utilization projects, such as biogas to electricity systems, since the early 2000’s. However, many projects were not able to sustain themselves because of the imbalance of demand and supply and cost and benefit. Financial incentives were crucial to the economic feasibility of current AD systems that utilize biomass, especially investment tax credits and federal grants [52]. In Italy, the use of financial incentives related to the production of electricity from renewable sources is not the highest, but has the fastest access [51].

These cost and benefit calculations were made based on well-defined input parameters from the literature or experimental results. Therefore, changes to the input parameters, such as the type of feedstock used, may affect the analysis.

## 5. Conclusions

This study aimed to evaluate the technical and economic feasibility of a co-AD plant as a decentralized solution for energy production. The behavior of two different types of feedstock has been evaluated: OFMSW made of FW and YW (Scenario 1) and a mixture of OFMSW and SS (Scenario 2). Five different levels of district extensions have been considered. Technical and economic parameters have been evaluated and compared to the current scenario, based on collection and composting of organic waste (Scenario 0). The obtained results show how all of the proposed scenarios are better than the reference scenario. With the rising of the district extension (as expressed by Nc), the co-AD plant increased the input and output streams. The introduction of SS in OFMSW produced a W_in_ stream characterized by a lower methane yield and VS content, but lower than 40% of TS. For both Scenario 1 and 2, the technical feasibility was ensured for all district cases. Despite the little amount of SS compared to the OFMSW one, changes to the AD process and waste properties were not negligible. The lack of biogas storage led to the adoption of CHP units of high-capacity values with long working time. Globally, Scenario 1 resulted in being the preferred path for organic waste. The introduction of SS in OFMSW increased costs, PBT, and the LCOW, other than generating a higher input and output waste amount and lower biogas yield. The feasibility of mono- and co-AD plants seemed to be effective in a medium district extension (about 15 condominiums) or larger (over 30 condominiums). For both 15 and 30 Nc, the economic analysis showed optimal results in terms of cumulated net costs, PBT, and the LCOW. The adoption of a mono-AD plant in a medium-scale district context was preferred. Indeed, this solution could lead the user to independently deliver the organic waste to the plant more easily, reducing the economic and environmental costs referred to the collection and transportation. Capital cost strongly affected the economic analysis, but revenue from the city for the management operation of the organic waste could significantly decrease costs. Financial incentives from governments could additionally help the growth of a local AD plant. The adoption of mono-AD plants in medium-sized urban districts of large cities could be a potentially excellent solution for OFMSW management in technical and economic terms. Further studies on the differences in the type of feedstock or environmental analysis through the life cycle assessment approach are considered necessary.

## Figures and Tables

**Table 1:** Building and demographic characteristics of the condominium.

Parameter	Symbol	Value
Number of units per condominium	Nu	24
Number of floors per unit	Nf	6
Number of apartments per floor	Na	3
Number of people per apartment	Np	2.6

**Table 2:** Parameters assumed for the characterization of SS and the calculation of SS_in_.

SS	X_i_	SSda_i_	TS	VS	Bulk Density	BMP	Methane Content
Units	adim.	kg/capita/d	[%]	[%TS]	[kg/l]	[mL CH_4_/g VS]	[%]
**Np_TOT_ < 10,000**							
PS	1	0.050	7.5	70	1.1	300	62
Ref.		0.050	7.4	72.9		243	62.2
		[23]	[24]	[24]		[24]	[24]
WAS	0						
**Np_TOT_ > 10,000**							
PS	1	0.050	25	70	1.6	125	62
Ref.		0.050	18.5	60.5		127	61.8
		[23]	[25]	[25]		[25]	[25]
WAS	1	0.025	15	75	1.3	250	67
Ref.		0.025	14.3	56.7	1.28	248.8	65.7
		[23]	[26]	[26]	[27]	[28]	[26]

**Table 3:** Characterization of organic substrates present in the literature in terms of TS and VS content, biogas, methane yield, and methane content.

Substrate	Description	TS [%]	VS [%TS]	Biogas Yield [mL Biogas/gVS]	CH_4_ Content [%]	BMP [mL CH_4_/gVS]	Ref.
OFMSW		8.6	73.5	454.3	58	261.4	[29]
FW	From UK kitchen	27.7	88.1	1035.5	62	642	[2]
OFMSW	Source sorted from house, paper wrapping, screened	26–31	87–91	496.7–801.6	60–61	298–489	[30]
OFMSW	Source sorted from apartment, paper wrapping, screened	28	87	781.3	64	500	[30]
OFMSW	Source sorted from apartment, plastic wrapping, screened	30–34	80–87	651.6–858.3	60–62	404–515	[30]
OFMSW	Source sorted from house, plastic wrapping, screened	30–33	81–84	669.0–924.2	58–62	388–573	[30]
OFMSW	Source sorted from house, paper wrapping, shredded	28	92	785.7	63	495	[30]
FW		28	86.1	657	67	353	[31]
YW	Blend of grass, leaves and branches	50.4	92	-	-	143	[3]
FW		26.6	93.4	-	-	560	[32]
YW		97.3	91.1	228.9	50.9	116.5	[12]
FW		23.9	91.3	862.2	61.1	526.8	[12]
OFMSW	YW + FW (3:1)	79.0	91.2	271.2	61	165.4	[12]
OFMSW	YW + FW (2:2)	60.6	91.2	466.1	63.5	296.0	[12]
OFMSW	YW + FW (1:3)	42.2	91.3	561.6	64.1	360.0	[12]
Mixture	YW + FW + SS (9:3:4)	63.4	82.8	268.7	61.3	164.7	[12]
Mixture	YW + FW + SS (6:6:4)	49.7	82.8	363.7	63.9	232.4	[12]
SS		30.1	90.1	518.2	66	342	[33]
SS	Dewatered	16.9	57.6	-	-	254.6	[12]
SS	Undewatered mixture of PS and WAS	3.5	65.7	-	-	248.8	[28]
PS	Undewatered	4.8	66.2	-	-		[34]
WAS	Undewatered	2.6	65.2	-	-		[34]
SS	Undewatered	3.0–3.1	70–74	-	-	260–460	[35]
SS	Dewatered	12.6	67.4	-	-	142	[36]
SS	Dewatered	17.7	67.2	-	-	173.1	[37]
SS	Dewatered mixture of PS and WAS	24.2	78.1	-	-	169	[38]
SS	Undewatered mixture of PS and WAS	3.3	84	514.8–643.9	64.3	331–414	[39]
WAS	Dewatered	4	72	120	66.6	80	[40]
WAS	Undewatered	0.7	73	390.7	62.2	243	[24]
WAS	Undewatered	1.5	67	536.1	72	386	[41]
WAS	Undewatered	1	72	585.8–726.5	61.8	363–449	[39]
PS	Undewatered	4.8–5.5	77–78	458.3	72	330	[39]
SS		20.6	51.5			280.4	[42]
PS	Undewatered	3	67	394.1	67.5	266	[40]

**Table 4:** Parameters assumed for the characterization of OFMSW.

OFMSW	TS	VS	Bulk Density	BMP	Methane Content
Units	[%]	[%TS]	[kg/L]	[mL CH_4_/g VS]	[%]
OFMSW	40.3	88.6	0.43	362.7	61.4
Ref.	42.2	91.3	0.27–0.55	360.0	60–61
	[12]	[12]	[21]	[12]	[30]

**Table 5:** The main parameters of the co-AD plant, dewatering system, and CHP unit.

Parameters	Units	Value	Ref.	
**co-AD Plant**				
HRT	d	30	22	[26]
T	°C	35	35	[4]
VS removal	%VS	90	92	[42]
Electrical self-consumption	kWh/t	60	60	[48]
Thermal self-consumption	kWh/t	40	37.6	[32]
**Dewatering System**				
H_2_O removal	%H_2_O	30	25–30	[44]
Electrical self-consumption	kWh/t	10	8.85	[49]
**CHP Unit**				
LHV CH_4_	MJ/m_3_	35.25	35	[50]
Power	kW	200	100–500	[51]
η_el_	%	35	39	[52]
η_th_	%	50	45	[52]
Electrical self-consumption	kWh/t	8	7.5	[32]

**Table 6:** Inputs for LCOW calculation.

Parameters	Units	Value		Ref.
Discount rate r	%	4	6	[51]
Lifetime t	y	20	20	[53]
Capital cost C	€/kW	4000	3789	[33]
Operative cost O	€/t	105	100	[29]
Treatement cost T	€/t	75	80	[55]
	€/capita	10	9.8	[1]
Electrical revenue Re	€/kWh	0.2	0.17–0.22	[51]
Thermal revenue Rt	€/kWh	0.045	0.12	[33]

**Table 7:** Results of the W_in_ streams properties for Scenario 1 (S1) and Scenario 2 (S2).

Nc	OFMSW [%]		TS [%]		VS [%TS]		Density [kg/l]		BMP [mL CH_4_/gVS]		CH_4_ Content [%]	
	S1	S2	S1	S2	S1	S2	S1	S2	S1	S2	S1	S2
5	100	92.3	40.3	37.8	88.6	87.2	0.43	0.48	362.7	357.9	61.4	61.5
10	100	92.3	40.3	39.2	88.6	87.2	0.43	0.52	362.7	344.4	61.4	61.5
15	100	88.9	40.3	38.3	88.6	86.7	0.43	0.55	362.7	340.9	61.4	61.7
20	100	88.9	40.3	38.3	88.6	86.7	0.43	0.55	362.7	340.9	61.4	61.7
30	100	88.9	40.3	38.3	88.6	86.7	0.43	0.55	362.7	340.9	61.4	61.7

**Table 8:** Results of W_in_, W_out_, B_out_, and V values for Scenario 1 (S1) and Scenario 2 (S2).

Nc	W_in_ [kg/d]		W_out_ [kg/d]		B_out_ [m^3^/d]		V [m^3^]	
	S1	S2	S1	S2	S1	S2	S1	S2
5	3370	3650	1683	1887	711	700	284	274
10	6739	7301	3366	3727	1422	1396	568	508
15	10,109	11,372	5049	5889	2133	2085	852	749
20	13,478	15,163	6732	7852	2844	2781	1136	999
30	20,218	22,745	10,098	11,779	4266	4171	1704	1498

**Table 9:** Results of nE_el_, nE_th_, gE_loss_, and self-consumption energy values for Scenario 1 (S1) and Scenario 2 (S2).

Nc	nE_el_ [kWh/d]		nE_th_ [kWh/d]		gE_loss_ [kWh/d]		Self-Consumption [kWh/d]	
	S1	S2	S1	S2	S1	S2	S1	S2
5	1232	1189	2001	1960	641	632	398	431
10	2465	2369	4002	3905	1282	1259	795	861
15	3697	3517	6003	5836	1922	1887	1193	1342
20	4929	4689	8005	7782	2563	2516	1590	1789
30	7394	7033	12,007	11,672	3845	3775	2386	2684

**Table 10:** Results of Nh, N_CHP_, and PBT values for Scenario 1 (S1) and Scenario 2 (S2).

Nc	Nh [h]		N_CHP_ [–]		PBT [y]	
	S1	S2	S1	S2	S1	S2
5	7	7	1	1	17	19
10	15	15	1	1	9	10
15	22	22	1	1	6	7
20	15	15	2	2	9	11
30	22	22	2	2	6	7

## Data Availability

The data that support the findings of this study are available from the corresponding author, upon reasonable request.
